# Changes in psychopathology and peripheral inflammation in schizophrenia patients initiating treatment with second-generation antipsychotics: a post-hoc analysis

**DOI:** 10.3389/fpsyt.2025.1644428

**Published:** 2025-09-05

**Authors:** Michel Heil, Monika Edlinger, Falko Biedermann, Timo Schurr, Alex Hofer

**Affiliations:** Department of Psychiatry, Psychotherapy, Psychosomatics and Psychological Medicine, University Clinic of Psychiatry I, Medical University Innsbruck, Innsbruck, Austria

**Keywords:** schizophrenia, CRP (C-reactive protein), PANSS (Positive and Negative Syndrome Scale), NLR (neutrophil-to-lymphocyte ratio), MLR (monocyte-to-lymphocyte ratio), SII (systemic immune-inflammation index)

## Abstract

Neuroinflammation has been proposed as a potential factor in the pathogenesis of schizophrenia and has been suggested to be both a state and a trait measure of the disease. However, the results of previous studies are inconsistent. The aim of the present study was therefore to investigate whether peripheral inflammatory markers such as C-reactive protein (CRP), the neutrophil-to-lymphocyte ratio (NLR), the monocyte-to-lymphocyte ratio (MLR), and the systemic immune-inflammation index (SII) can reliably indicate disease severity in schizophrenia. 116 (52.6% male) cases diagnosed with schizophrenia commencing monotherapy with a second-generation antipsychotic were included in this study. Next to baseline evaluation of sociodemographic and clinical data, the Positive and Negative Syndrome Scale (PANSS) was used at the beginning and after 2, 4, 8, 12, and 24 weeks of treatment. Blood samples were collected simultaneously to measure CRP levels, NLR, MLR, and SII. Linear mixed-effects models were used to investigate whether PANSS (sub)scores and inflammatory markers changed over time and whether they were associated with each other. We found a significant reduction of most PANSS (sub)scores from baseline to follow-up measurements, while CRP levels, NLR, MLR, and SSI did not change between measurements. Results did not show statistically significant associations between PANSS (sub)score changes and changes in markers of neuroinflammation. This was also true when controlling for sex, age, smoking status, and body-mass index. This study found no evidence of an association between the levels of peripheral inflammatory markers and disease severity in schizophrenia.

## 1 Introduction

There is a growing body of literature suggesting a relationship between neuroinflammation and schizophrenia (1), but findings have been inconsistent (2–4). Several studies have focused on different inflammatory biomarkers like cytokines (5, 6) or acute-phase reactants like C-reactive protein (CRP) (7), the latter showing some advantages as a biomarker of systemic inflammation (8). Sampling and analysis of CRP are simple, cost-effective, and precise. It is easily detected in serum (9), has relatively low within-person variability, shows no diurnal variation, and meets World Health Organization standards (10). Numerous previous studies have investigated the relationship between CRP levels and schizophrenia and have come to contradictory conclusions (7, 11, 12). For instance, Joseph and colleagues (13) reported that CRP did not correlate with the severity of positive or disorganized symptoms of the disorder, cognitive function, age of onset, or duration of illness but was associated with physical and metabolic (body-mass-index [BMI], glucose, HbA1c) factors. In turn, Fernandes et al. (7) found that peripheral CRP levels increased in proportion to the progression of positive symptoms. In a recent review, Fond et al. (14) considered CRP to be a useful screening marker for detecting inflammation in schizophrenia. Other authors go even further by considering CRP as a biomarker of disease activity (7, 15). In line with this consideration, several studies in different cultural and ethnic groups found significantly higher CRP levels in patients with schizophrenia compared to healthy control subjects (13, 16–21), especially in those treated with antipsychotic medication (22). However, it remains unclear whether this is a direct drug effect or related to factors like an elevated BMI (7, 23). Notably, randomized clinical trials have shown improvements in psychotic symptoms following initiation of adjuvant anti-inflammatory treatment (24–26), however, the findings of these studies are limited by small sample sizes (27) and conflicting results (26) and long-term observations are missing (25).

Next to cytokines and CRP, cell counting and ratios such as the neutrophil-to-lymphocyte ratio (NLR), the monocyte-to-lymphocyte ratio (MLR), and the systemic immune-inflammation index (SII) which use a simple calculation based on peripheral lymphocyte, neutrophil, and platelet counts, have the potential to sensitively reflect inflammatory response and are considered prognostic markers in a variety of diseases (28–35). High MLR, for example, has recently been shown to represent an independent risk factor for post-stroke depression, implying that it might be involved in post-stroke depression inflammatory mechanisms (36). Similarly, high NLR has also been associated with post-stroke depression (37) and has also been found in patients with schizophrenia (38, 39). This corroborates the findings of a recent meta-analysis showing that individuals with non-affective psychosis have significantly higher NLR and MLR compared to healthy control subjects (40). However, knowledge about a potential association between SII and non-affective psychoses is limited. In a recent study, both patients with anti-NMDAR encephalitis and first-episode schizophrenia were found to have elevated SIIs compared to healthy controls, again supporting the hypothesis of a link between schizophrenia and the immune system (41). Chen et al. (42) found this link in the context of cognitive symptoms in schizophrenia: Patients with cognitive impairment have higher NLR, platelet-to-lymphocyte ratio (PLR), and SII compared to those without deficits, whereas lymphocytes were considerably positively correlated with cognitive function.

Taking into account the above mentioned conflicting results of previous studies the current *post-hoc* analysis aimed to clarify whether a general peripheral inflammatory marker could serve as a reliable measure of disease severity among chronically ill schizophrenia patients starting treatment with second-generation antipsychotics. Accordingly, we hypothesized that symptom changes occurring during the course of treatment would be associated with changes in one or more peripheral inflammatory markers (CRP, NLR, MLR, and SII).

## 2 Materials and methods

### 2.1 Sample

From October 1997 to September 2010 schizophrenia patients aged between 18 and 65 years who were treated in an in- or outpatient unit at the Department of Psychiatry, Psychotherapy, Psychosomatics and Medical Psychology of the Medical University Innsbruck were allocated to a prospective, naturalistic drug monitoring program when starting monotherapy with a second-generation antipsychotic drug. Diagnoses according to ICD-10 were confirmed by chart information and reports from referring clinicians. The analysis of data was conducted from October 2023 to May 2024. The study was approved by the Ethical Committee of the Medical University Innsbruck on September 25^th^, 1997 (Study number: AM742b) and all patients gave written informed consent for study participation.

Patients who had previously been taking antipsychotic medications and had to switch them because of insufficient efficacy or intolerance were included into the study after a wash-out period of 3–5 days in those with oral treatment and one injection interval in those on long-acting injectable medication. Patients undergoing more than one treatment episode in the course of the study were included more than once following the same procedure and receiving monotherapy with different antipsychotics in each of the treatment periods. The type of antipsychotic treatment was chosen by the treating physician. Dosing was within the recommended ranges and followed clinical needs. Drug-naïve individuals experiencing a first episode of the illness were not included in these analyses because significant differences in CRP levels in this group compared to chronically ill patients have been described (21). Next to antipsychotic treatment the use of benzodiazepines (treatment of agitation, anxiety, and sleep disorders), biperiden/propranolol (treatment of extrapyramidal motor symptoms, akathisia, and hypersalivation), as well as antidepressants and mood stabilizers were tolerated. Individuals with anti-inflammatory medication were excluded from the analyses. No patient was treated with an antibiotic during the participation in the study.

### 2.2 Experimental design

Sociodemographic and clinical data were collected at baseline. Psychopathology was rated at baseline and after 2, 4, 8, 12, and 24 weeks of treatment using the PANSS (43). Patient data obtained between week 11 and 14 was labelled as *week 12* assessment, patient data from week 24 until week 28 was labelled as *week 24* assessment. According to Chen and coworkers (44) the PANSS was divided into four dimensions representing positive, negative, affective, and cognitive symptoms. Ratings were conducted by psychiatrists belonging to a trained schizophrenia research team. Blood samples were taken at the same points in time. Apart from other laboratory values full blood count and CRP levels were measured by the ISO 9001-certified Central Institute of Medical and Chemical Laboratory Diagnostics of the University Hospital of Innsbruck. Until October 2003, blood counts were measured on the Beckman Coulter STKS hematology instrument and on the Sysmex XE-2100 thereafter. Both instruments use flow cytometry. At the time the study was conducted, a CRP threshold value of 0.5 mg/dL was standard in our hospital’s laboratories. SII is defined as follows: SII = P × N/L (P, N, L: platelet counts, neutrophil counts, and lymphocyte counts) (29).

### 2.3 Statistical methods and analysis

Cases with at least two assessment times and a baseline value of full blood count, CRP level, and PANSS score were included in the statistical analysis.

The primary aim of the statistical analysis was the illustration of the relation between changes in peripheral inflammatory marker levels (CRP, NLR, MLR, and SII) and changes in PANSS (sub)scores. In addition, we were interested in how these values were related to each other at each measurement and whether changes occurred during the course of treatment. Changes in PANSS illness severity were evaluated at Baseline, Week 2, Week 4, Week 8, Week 12, and Week 24, categorizing patients according to Leucht et al. (45) as mildly, moderately, markedly, severely, or extremely ill. Improvement, deterioration, or no change in severity between time points was summarized in **Appendix Table 4**. CRP levels were compared across illness severity categories, with descriptive statistics presented for each time point in **Appendix Table 5**.

For statistical analyses R (version 4.2.0) and the nlme package (version 3.1-164) were used. Statistical tests were two-tailed. The significance level alpha was set to 5%. Sociodemographic and clinical data were reported by mean, standard deviation, and counts. Initially, a linear mixed-effects model was used to investigate whether PANSS scores and inflammatory markers changed over time compared to baseline values. Subsequently, a second model was constructed to examine sex differences by including sex as an independent variable. The primary analysis was conducted using a similar model to account for both fixed and random effects. The dependent variable was the difference score on the PANSS from baseline to the respective follow-up measurement. The primary independent variables were similarly calculated with difference values for CRP, NLR, MLR, and SII. Additionally, we compared models including sex, age, smoking status, and BMI as covariates. A random intercept was included to capture the variability among cases and an autoregressive covariance structure (AR[1]) was implemented to account for the correlation between repeated measures within cases over time. Model diagnostics were performed to check for violations of model assumptions, including normality of residuals and homoscedasticity. The goodness-of-fit was assessed using the Akaike Information Criterion (AIC) and Bayesian Information Criterion (BIC). Spearman correlations between the variables of interest were created for a detailed presentation of the relationships.

To improve the validity of our findings: Participants with CRP values indicative of acute inflammation (CRP > 1 mg/dL) at baseline or at any subsequent assessment were excluded from all analyses, as recommended by Pagana et al. (46) and Singh et al. (47) and recent literature on chronic peripheral inflammation in schizophrenia (48). This step was taken to minimize the confounding effects of acute inflammatory states on the interpretation of chronic peripheral inflammation markers (CRP, SII, NLR, and MLR). The number of cases excluded due to elevated CRP at each time point is reported in **Appendix Table 6**. The primary analysis was re-conducted following these exclusions (**Appendix Table 7**).

## 3 Results

### 3.1 Sample characteristics

Demographic and clinical characteristics of the study sample are summarized in **Table 1**. Data of 116 cases were available for baseline analysis (T0). They had a mean age of 35.8 years (SD = 10.3) and a mean duration of illness of 8.5 years (SD = 7.2). Sex distribution was balanced (male: 52.6%; female: 47.4%). More than half (53.6%) of study participants were smokers, mean BMI was 26.6 ± 6.2 with a range from 17.6 to 45.2 (underweight to obese class III). 25 cases (21.6%) showed an elevated concentration of CRP and five of these cases also showed elevated leucocyte concentrations (>10,000 cells/µl). Baseline total scores of the Positive and Negative Syndrome Scale (PANSS) (43) ranged from 31 to 128 with a mean value of 66.9 (SD = 21.2). **Table 1** provides an overview of the antipsychotic compounds prescribed and the frequency of prescription.

**Table 1:** Sociodemographic and clinical variables at baseline (n=116 cases).

Variable	Mean ± SD or N (%)
Age (years)	35.8 ± 10.3
Sex	
Male	61/116 (52.6%)
Female	55/116 (47.4%)
Psychiatric treatment	
Outpatient unit	30/116 (25.9%)
Inpatient unit	43/116 (37.1%)
Combined outpatient and inpatient units	31/116 (26.7%)
Smokers	59/116 (53.6%)
BMI (kg/m²)	26.6 ± 6.2
Duration of illness (years)	8.5 ± 7.2
CRP (mg/dl)	0.47 ± 0.58
Elevated CRP concentration (> 0.5 mg/dl)^a^	25/116 (21.6%)
NLR	1.92 ± 0.93
MLR	0.26 ± 0.13
SII	474.6 ± 259.2
Symptomatology	
PANSS total score	69.9 ± 21.2
PANSS factors (Chen et al. (44); range 1–7)	
Positive	2.53 ± 1.27
Negative	2.57 ± 0.98
Affective	2.51 ± 0.85
Cognitive	2.04 ± 0.76
Newly initiated antipsychotic drug	
Amisulpride	21/116 (18.1%)
Aripiprazole	12/116 (10.3%)
Clozapine	21/116 (18.1%)
Olanzapine	18/116 (15.5%)
Quetiapine	11/116 (9.5%)
Risperidone	13/116 (11.2%)
Sertindole	8/116 (6.9%)
Ziprasidone	10/116 (8.6%)
Zotepine	2/116 (1.8%)

a Among the cases with elevated CRP concentration, 5 had elevated leucocyte concentration (> 10,000), 20 had normal leucocyte values (<10,000).

BMI, body-mass-index; CRP, C-reactive protein; SII, systemic immune-inflammation index; NLR, neutrophil-to-lymphocyte ratio; MLR, monocyte-to-lmyphocyte ratio; PANSS, Positive and Negative Syndrome Scale.

Data of 57 cases were available for analysis at weeks 2 (T1) and 4 (T2), of 26 cases at week 8 (T3), of 45 at week 12 (T4), and of 26 at week 24 (T5). The time course of symptomatology and mean inflammatory marker levels are shown in **Table 2**. The PANSS total score decreased significantly between baseline measurement (T0, mean = 69.9, SD = 21.2) and all consecutive measurements (T2 to T5; mean_t2_ = 59.0 to mean_t5_ = 52.7), with no significant differences between the sexes (p > 0.05). In terms of the Chen et al. (44) four factor model, positive, affective, and cognitive symptoms improved significantly from baseline to consecutive follow-ups, whereas the degree of negative symptoms remained unchanged over time except for the difference between baseline and week 12 of treatment. Over the observation period, mean CRP levels, NLR, MLR, and SII did not change significantly (all p > 0.05). NLR was the only exception with significantly (p = 0.0443) increased mean levels in week 4 (T2, mean = 2.47, SD = 3.35) compared to baseline measurement (mean = 1.92, SD= 0.93). Again, no significant differences were found between the sexes in terms of inflammatory markers (all p > 0.05).

**Table 2:** Time course of symptomatology and inflammatory markers’ mean concentration.

		PANSS total score	PANSS positive	PANSS negative	PANSS affective	PANSS cognitive	CRP	SII	NLR	MLR
Baseline (mean)		69.9	2.53	2.56	2.51	2.04	0.47	475.1	1.92	0.26
SD		21.2	1.27	0.98	0.85	0.76	0.58	259.2	0.93	0.13
N		114	114	115	115	115	116	107	107	107
Week 2 (mean)		59.0	1.96	2.32	2.02	1.74	0.55	513.4	2.04	0.27
SD		20.0	1.01	0.96	0.73	0.67	0.85	419.3	1.48	0.14
N		56	55	56	56	56	57	55	55	55
inear mixed-effects model (Week 2 vs. Baseline)	t	-5.23	-5.40	-1.84	-6.06	-3.60	-1.07	0.45	0.43	0.31
	*p-value*	<0.0001	<0.0001	0.0678	<0.0001	0.0003	0.2884	0.6507	0.6703	0.7556
Week 4 (mean)		60.0	1.89	2.45	2.11	1.73	0.48	614.7	2.47	0.33
SD		19.3	0.91	0.95	0.84	0.60	0.43	877.2	3.35	0.57
N		55	55	55	55	55	57	52	52	52
Linear mixed-effects model (Week 4 vs. Baseline)	t	-5.71	-6.00	-1.59	-5.79	-4.72	-0.21	1.94	2.02	1.75
	*p-value*	<0.0001	<0.0001	0.1135	<0.0001	<0.0001	0.8343	0.0533	0.0443	0.0819
Week 8 (mean)		54.3	1.72	2.17	1.82	1.62	0.48	568.2	2.10	0.27
SD		19.9	0.95	0.93	0.71	0.63	0.46	336.4	0.82	0.10
N		26	26	26	26	26	26	26	26	26
Linear mixed-effects model (Week 8 vs. Baseline)	t	-5.15	-5.11	-1.87	-5.60	-3.86	0.03	0.67	0.41	0.11
	*p-value*	<0.0001	<0.0001	0.0622	<0.0001	0.0002	0.9793	0.5067	0.6843	0.9093
Week 12 (mean)		53.9	1.50	2.30	1.84	1.58	0.54	544.3	2.15	0.24
SD		18.4	0.55	1.03	0.73	0.58	0.63	294.0	1.00	0.08
N		44	43	44	44	44	45	42	42	42
Linear mixed-effects mode (Week 12 vs. Baseline) l	t	-6.69	-7.08	-2.46	-7.04	-4.77	0.73	0.68	0.67	-0.41
	*p-value*	<0.0001	<0.0001	0.0145	<0.0001	<0.0001	0.4652	0.4979	0.5009	0.6807
Week 24 (mean)		52.7	1.45	2.36	1.77	1.49	0.54	540.8	2.05	0.26
SD		15.6	0.79	0.84	0.67	0.51	0.68	246.4	0.77	0.10
N		26	26	26	26	26	26	26	26	26
Linear mixed-effects model (Week 24 vs. Baseline)	t	-5.60	-6.67	-0.56	-6.62	-4.56	0.77	0.31	0.28	-0.09
	*p-value*	<0.0001	<0.0001	0.5754	<0.0001	<0.0001	0.4416	0.7560	0.7784	0.9282

CRP, C-reactive protein; SII, systemic immune-inflammation index; NLR, neutrophil-to-lymphocyte ratio; MLR, monocyte-to-lmyphocyte ratio; PANSS, Positive and Negative Syndrome Scale.

### 3.2 Association of changes in peripheral inflammatory markers with changes in PANSS scores

Model diagnostics indicated that the model assumptions were satisfactorily met. Several models were analyzed, adjusting for different covariates including sex, age, smoking status, and BMI. The results of the linear mixed-effects models (**Table 3**) indicated no significant association between changes in CRP levels and changes in PANSS scores in models without adjustment (model 1: estimate = -1.327, SE = 0.994, p = 0.1836) and models adjusting for sex, age (model 2: estimate = -1.236, SE = 0.995, p = 0.2156), and smoking status (model 3: estimate = -1.253, SE = 0.999, p = 0.2113). However, a significant association was found when additionally adjusting for BMI (model 4: estimate = -2.146, SE = 1.045, p = 0.0415). No significant associations were found between changes in SII, NLR, or MLR and changes in PANSS scores across all unadjusted models (all p > 0.05). The inclusion of the above mentioned covariates did not change the overall non-significance of the results (all p > 0.05).

**Table 3:** Linear mixed-effects analysis investigating the association of changes in peripheral inflammatory marker levels and changes in PANSS scoring using different adjusted models.

Model			Estimate	S.E.	df	t	Estimate (95% CI)		*p-value*	AIC	BIC
							LB	UB			
CRP	Model 1	CRP	-1.327	0.994	211	-1.334	-3.287	0.634	0.1836	2434	2453
	Model 2	CRP (adjusted for sex & age)	-1.236	0.995	210	-1.242	-3.200	0.726	0.2156	2434	2461
	Model 3	CRP (adjusted for sex, age, & SS)	-1.253	0.999	206	-1.254	-3.224	0.717	0.2113	2367	2397
	Model 4	CRP (adjusted for sex, age, SS, & BMI)	-2.146	1.045	188	-2.053	-4.207	-0.084	0.0415	2195	2228
SII	Model 1	SII	< -0.0001	0.001	193	-0.072	-0.002	0.002	0.9425	2266	2284
	Model 2	SII (adjusted for sex & age)	< -0.0001	0.001	192	-0.062	-0.002	0.002	0.9507	2267	2293
	Model 3	SII (adjusted for sex, age, & SS)	< -0.0001	0.001	188	-0.007	-0.002	0.002	0.9941	2206	2236
	Model 4	SII (adjusted for sex, age, SS, & BMI)	< -0.0001	0.001	170	-0.203	-0.002	0.002	0.8397	2038	2070
NLR	Model 1	NLR	-0.110	0.312	193	-0.351	-0.726	0.506	0.7258	2254	2273
	Model 2	NLR (adjusted for sex & age)	-0.107	0.313	192	-0.342	-0.723	0.510	0.7330	2256	2281
	Model 3	NLR (adjusted for sex, age, & SS)	-0.100	0.313	188	-0.318	-0.718	0.518	0.7506	2195	2224
	Model 4	NLR (adjusted for sex, age, SS, & BMI)	-0.133	0.330	170	-0.402	-0.784	0.519	0.6883	2026	2059
MLR	Model 1	MLR	0.708	1.957	193	0.362	-3.151	4.567	0.7179	2251	2269
	Model 2	MLR (adjusted for sex & age)	0.818	1.960	192	0.417	-3.048	4.684	0.6768	2252	2278
	Model 3	MLR (adjusted for sex, age, & SS)	0.817	1.961	188	0.417	-3.052	4.686	0.6773	2191	2221
	Model 4	MLR (adjusted for sex, age, SS, & BMI)	0.872	2.090	170	0.417	-3.253	4.997	0.6769	2023	2055

AIC, Akaike Information Criterion; BIC, Bayesian Information Criterion; BMI, body-mass-index; CRP, C-reactive protein; df, degree of freedom; LB, lower bound; MLR, monocyte-to-lymphocyte ratio; NLR, neutrophil-to-lymphocyte ratio; S.E., standard error; SII, systemic immune-inflammation index; SS, Smoking status; UB, upper bound.

### 3.3 Correlation of symptomatology with CRP, NLR, MLR, and SII

Regarding the simultaneous assessment of symptomatology and peripheral inflammatory markers, the results of Spearman rank correlation analyses revealed no statistically significant association between PANSS (sub)scores and CRP, NLR, MLR, and SII at any time of investigation after correction for multiple testing (Bonferroni) (**Appendix Table 1**). Exclusion of cases with increased leucocytes left these findings unchanged. The associations of changes in PANSS (sub)scores with changes in inflammatory marker concentration are depicted in **Appendix Table 2**. Again, we found no significant correlation between changes in the PANSS total score and PANSS factors according to Chen et al. (44) and changes in CRP, NLR, MLR, and SII at any assessment time. Exclusion of cases with increased leucocytes as well as differentiating cases who were considered to be mildly or moderately ill at baseline (i.e. PANSS total score ≤75) (45) from the remaining study participants left these findings unchanged.

### 3.4 Correlation of inflammatory markers with age, sex, BMI, and smoking status

Both age and BMI were positively associated with CRP concentration at T0, T1, T2, T3, and T4 (moderate to strong correlation). After six months of treatment (T5), a higher CRP concentration correlated moderately positively with female sex and older age, however, statistical significance vanished after correction for multiple testing. NLR, MLR, and SII did not persistently correlate with age, BMI, sex, or smoking status. NLR and SII were positively associated with age and BMI at T1 (moderate correlation), whereas MLR correlated strongly with male sex at T5 (see **Appendix Table 3**).

### 3.5 Exclusion of cases with acute inflammation

In line with recent recommendations and reference laboratory values, we excluded cases with CRP values > 1 mg/dL at baseline or any follow-up assessment, as these indicate acute inflammation and could confound the assessment of chronic peripheral inflammation. The number of excluded cases per time point is summarized in **Appendix Table 6**. Overall, less than one third of cases were excluded at each assessment (range: 3.9% to 26.3% of cases per time point). After exclusion of these cases, the main results regarding the association between inflammatory markers and symptomatology remained unchanged (**Appendix Table 7**).

## 4 Discussion

The present investigation did not reveal any statistically significant associations between longitudinal changes in CRP levels, NLR, MLR, or SII and PANSS (sub)score changes in chronically ill schizophrenia patients initiating treatment with a second-generation antipsychotic drug. This finding is partially consistent with a previous meta-analysis that found no association between CRP levels and PANSS total scores (7). However, in contrast to our findings, that meta-analysis identified a linkage between increased peripheral CRP levels and the severity of positive symptoms as assessed by the PANSS – a finding that aligns with the observations made by Steiner and colleagues (21). This is not reflected in our results and may be related to the relatively low mean PANSS positive symptoms subscore in our sample.

Although a number of studies have consistently reported elevated CRP levels in schizophrenia patients compared to healthy controls (49) the significance of this finding remains unclear. The question arises as to whether they are merely by-products of the pathogenesis of schizophrenia or whether they could play a critical role in the clinical manifestation of the disorder (50). On the other hand, Hartwig and coworkers (27) even suspected a protective effect of CRP on the risk of schizophrenia.

A recent study revealed that NLR was significantly higher in patients with schizophrenia compared to a healthy control group. Intriguingly, this study found no significant relationship between NLR and various clinical parameters in patients, including the number of hospitalizations, duration of illness, or illness severity as assessed by the Clinical Global Impression-Severity Scale (CGI-S) (51) and the PANSS. Furthermore, no correlation was found between NLR and other inflammatory and metabolic laboratory values such as fasting blood glucose, insulin, HbA1c, triglycerides, total cholesterol, and CRP (52). These and other conflicting findings (38, 53–55) reinforce the need to further explore the likelihood of inflammatory response in the context of schizophrenia.

The heterogeneity observed in different studies could be due to the inherent variability of schizophrenia, including differences between the different stages of illness and the specific subgroups studied. For example, higher CRP levels have been observed in schizophrenia patients with depressive symptoms (56) and correlations between NLR and illness severity, as assessed by the Brief Psychiatric Rating Scale (BPRS) (57) or the CGI-S, were also more pronounced in unmedicated patients than in chronically ill people undergoing antipsychotic treatment (55). Such nuances call for a more differentiated approach in future research.

Our linear mixed-effects model analysis revealed no significant associations between changes in CRP levels and changes in PANSS scores in unadjusted models. This finding remained unchanged when adjusting for sex, age, and smoking status. However, a significant association was found when additionally adjusting for BMI, which is consistent with the results of a recent meta-analysis (58). In turn, no significant associations were found between changes in SII, NLR, or MLR and changes in PANSS scores across all models. These results suggest that BMI may have an impact on the relationship between CRP levels and symptom severity, however, the overall impact of peripheral inflammatory markers on schizophrenia symptoms remains unclear. Alternative inflammatory biomarkers may thus be more appropriate to investigate this issue. For example, Del Giudice and Gangestad (9) reported that CRP can be elevated even in the complete absence of an inflammatory state. They therefore recommended the use of other inflammatory biomarkers such as IL-1β or TNF-α. Similarly, ratios other than those calculated in the present study, such as the CRP/albumin ratio and the neutrophil/albumin ratio, as well as cytokines related to the kynurenine pathway like IFN-γ, IL-4, IL- 8, and IFN-α (59) may be more appropriate in this context and should be considered in future studies (60).

Our finding of a positive correlation between CRP and BMI is consistent with the results of previous studies in mentally healthy overweight and obese individuals (61–63) as well as in schizophrenia patients (64). Similarly, earlier studies have also described higher CRP levels in older people, which may be due to declining sex hormone levels and an increase in visceral adipose tissue with increasing age (65). However, this issue cannot be addressed by our data.

In general, women have higher CRP levels than men, a phenomenon that may be attributed to sex-specific fat distribution (66) and increased plasma leptin levels (67, 68). The fact that we could not detect any sex differences in our sample is most likely due to the large age range (18 to 63 years) and BMI variability (17.5 to 45.2), since both age and obesity influence CRP-levels in a sex-specific manner (61–63, 65–68). A recent investigation analyzing the correlation between age and CRP within a psychiatric inpatient population found comparable shifts in sex-specific CRP concentrations among patients diagnosed with schizophrenia spectrum disorder and those with unipolar depression (69). As inflammation is frequently observed in major depressive disorder (70) and schizophrenia (18), the effects of age, BMI, and/or sex may be modified by the overarching influence of diagnosis-related inflammation. It is likely that the inflammatory processes associated with schizophrenia may mask other effects.

In light of the complexity of peripheral inflammation in schizophrenia, recent findings from a systematic review and meta-analysis add another layer of understanding. This analysis of paired blood-cerebral spinal fluid (CSF) samples revealed a generally poor correlation between peripheral and central inflammatory markers (71), i.e. peripheral inflammatory markers may not accurately reflect neuroinflammatory events. Future research should therefore focus on identifying peripheral markers that accurately reflect inflammatory processes in the central nervous system and how they relate to schizophrenia.

Limitations of the present study warrant acknowledgment. Firstly, the absence of a control group restricted our ability to comparatively assess CRP levels, NLR, MLR, and SII. Although we adjusted the levels of peripheral inflammatory markers for several potential confounders such as BMI, age, sex, and smoking status, there remains a possibility that unknown or hidden confounders may be missed. Secondly, we focused on a potential associations between changes in symptomatology during treatment and the aforementioned inflammatory markers. Investigation of other acute phase reactants or inflammatory cytokines and biomarkers could potentially yield different results. Thirdly, while subgroup analyses based on illness severity or inpatient/outpatient status might have provided more nuanced insights, our sample size was not large enough to perform reliable stratified analyses without the risk of underpowering the results. Such analyses would have been exploratory, with a high risk of type II error. Nevertheless, for transparency, we have included **Appendix Tables 4** and **5**, which summarize changes in PANSS illness severity and CRP levels across illness severity categories, respectively. Lastly, this study included both inpatients and outpatients, which may have led to variations in inflammatory markers. However, the treatment setting does not necessarily provide information about the severity of the study participants’ illness, especially since patients were included in the study even if side effects of the medication required a change in therapy and inpatient admission was necessary for social or practical reasons. Moreover, the treatment setting could also change in the course of the study and accordingly, this variable could not be taken into account in our calculations. Notwithstanding these limitations, our investigation adds to the growing body of research on a possible association between peripheral inflammatory markers and symptomatology in schizophrenia. One key strength of our research is its reflection of clinical routine. In the clinical setting, peripheral blood sampling is performed routinely because it is simple, rapid, and minimally invasive. Conversely, CSF examinations provide a more direct insight into the neuroinflammatory status of the CNS, but are impractical in daily practice.

In summary, our real-world findings demonstrate that peripheral markers such as CRP, NLR, MLR, and SII do not accurately reflect symptom severity in patients with schizophrenia. While our study sheds light on certain aspects of the interface between inflammation and schizophrenia, it also highlights the complicated and multifaceted nature of this relationship. The inconsistencies between different studies indicate that more comprehensive research is urgently needed that takes into account both peripheral and central inflammatory markers.

## Data Availability

The raw data supporting the conclusions of this article will be made available by the authors, without undue reservation.
