# Supplementary material for: Changes in psychopathology and peripheral inflammation in schizophrenia patients initiating treatment with second-generation antipsychotics: a post-hoc analysis
Source: Front Psychiatry. 2025 Sep 5;16:1644428. doi: 10.3389/fpsyt.2025.1644428 (PMC12447443; doi:10.3389/fpsyt.2025.1644428)
Supplement: Supplementary file 1 [file DataSheet1.pdf]

Appendix Table 1: Correlation of CRP, SII, NLR, and MLR with PANSS (sub)scores (Spearman rank correlation)

		PANSS total score	PANSS positive	PANSS negative	PANSS affective	PANSS cognitive
Baseline	hs-CRP (N=116)	.137	.144	.054	.028	.102
	SII (N=107)	-.016	.046	-.188	.017	.026
	NLR (N=107)	-.089	.025	**-.255***	-.065	-.038
	MLR (N=107)	.057	.067	-.063	.086	.094
Week 2	hs-CRP (N=57)	-.028	.007	-.080	-.031	.013
	SII (N=55)	**-.312***[c]	-.153	**-.326***[c]	**-.310***[c]	-.228
	NLR (N=55)	-.211	-.091	-.218	-.243	-.128
	MLR (N=55)	.222	.146	.259	.141	**.286***[c]
Week 4	hs-CRP (N=57)	-.065	.015	-.175	-.027	-.073
	SII (N=52)	**-.290***[c]	-.146	-.211	-.243	**-.282***
	NLR (N=52)	-.267	-.162	-.164	-.257	-.240
	MLR (N=52)	.185	.191	.173	.163	.156
Week 8	hs-CRP (N=26)	-.129	-.051	-.194	-.155	-.118
	SII (N=26)	-.136	-.090	-.206	-.008	-.163
	NLR (N=26)	.079	.077	-.001	.121	.028
	MLR (N=26)	.266	.164	.209	.122	.351
Week 12	hs-CRP (N=45)	-.063	-.041	-.069	-.075	.007
	SII (N=42)	-.118	.147	-.267	-.054	.015
	NLR (N=42)	-.036	.181	-.138	-.005	.053
	MLR (N=42)	.115	.083	-.004	.121	.195
Week 24	hs-CRP (N=26)	-.200	.071	-.381	-.151	-.114
	SII (N=26)	.012	**.414***[c]	-.234	.207	-.067
	NLR (N=26)	.087	.376	-.121	.298	.019
	MLR (N=26)	.232	.311	.123	.318	.189

* $p \leq 0.05$

[c] No longer significant after correction for multiple testing (Bonferroni)

Abbreviations: CRP= C-reactive protein; SII=systemic immune-inflammation index; NLR=neutrophil-to-lymphocyte ratio; MLR=monocyte-to-lmyphocyte ratio; PANSS=Positive and Negative Syndrome Scale.

Appendix Table 2: Correlation of changes in CRP, SII, NLR, and MLR levels with changes in PANSS (sub)scores (Spearman Rank correlation)

		PANSS total score	PANSS positive	PANSS negative	PANSS affective	PANSS cognitive
Baseline to week 2	CRP change (N=57)	-.056	-.053	-.095	-.059	-.036
	SII change (N=55)	-.099	-.101	-.231	.083	-.008
	NLR change (N=55)	-.195	-.147	**-.286*[a]**	-.025	-.115
	MLR change (N=55)	-.177	-.159	-.156	-.064	-.065
Baseline to week 4	CRP change (N=57)	.112	-.023	-.022	-.027	.050
	SII change (N=52)	-.132	-.218	.005	-.167	-.027
	NLR change (N=52)	-.084	-.147	-.042	-.121	.079
	MLR change (N=52)	-.105	-.099	-.010	-.084	-.106
Baseline to week 8	CRP change (N=26)	.080	.002	.039	-.161	.074
	SII change (N=26)	-.069	.006	.110	-.176	-.013
	NLR change (N=26)	-.075	-.068	.161	-.230	.012
	MLR change (N=26)	.191	.351	.163	.290	.267
Baseline to Week 12	CRP change (N=45)	-.132	.125	.088	-.041	.052
	SII change (N=42)	-.235	-.191	-.154	-.162	-.148
	NLR change (N=42)	**-.326*[a]**	**-.338*[a]**	-.125	-.237	-.247
	MLR change (N=42)	.096	.109	-.202	**.306*[a]**	.091
Baseline to Week 24	CRP change (N=26)	.233	.123	.057	.006	.061
	SII change (N=26)	.206	.219	.008	.247	.074
	NLR change (N=26)	.108	.119	-.028	.191	-.038
	MLR change (N=26)	.278	.223	.108	.307	.277

* $p < 0.05$

[a] No longer significant after correction for multiple testing (Bonferroni)

Abbreviations: CRP= C-reactive protein; SII=systemic immune-inflammation index; NLR=neutrophil-to-lymphocyte ratio; MLR=monocyte-to-lmyphocyte ratio; PANSS=Positive and Negative Syndrome Scale.

Appendix Table 3: Correlation of CRP, SII, NLR, and MLR with age, sex, BMI, and smoking status

		Sex (female vs. male)	Age	BMI	Smoking
Baseline	CRP (N=110-116)	-.028	**.241**^{**b}	**.372****	.000
	SII (N=101-107)	.045	.144	.174	-.105
	NLR (N=101-107)	-.031	.120	.132	-.097
	MLR (N=101-107)	-.131	.007	.051	-.095
Week 2	CRP (N=52-64)	.039	**.452****	**.527****	.152
	SII (N=55)	**.298***^{b}	**.427****	**.408****	.067
	NLR (N=55)	.238	**.357****	**.295***^{b}	.002
	MLR (N=55)	-.042	-.129	-.093	-.114
Week 4	hs-CRP (N=55-68)	.097	**.332****	**.418****	.047
	SII (N=52)	.121	.148	.093	.019
	NLR (N=52)	.051	.104	.054	-.019
	MLR (N=52)	-.040	.238	-.037	.050
Week 8	CRP (N=48-49)	.159	**.387****	**.419****	.063
	SII (N=26)	.262	**.440***^{b}	.111	.048
	NLR (N=26)	.151	.377	.017	-.087
	MLR (N=26)	.000	-.098	-.361	-.368
Week 12	CRP (N=49-50)	.088	**.396****	**.401****	.032
	SII (N=42)	.228	.256	.049	-.167
	NLR (N=42)	.040	.123	.032	-.270
	MLR (N=42)	-.268	-.052	-.070	-.048
Week 24	CRP (N=30)	**.384**^{*a}	**.380**^{*b}	.270	-.094
	SII (N=26)	-.044	.199	.012	-.089
	NLR (N=26)	-.278	.229	-.009	-.211
	MLR (N=26)	**-.600****	-.043	-.186	-.256

* $p < 0.05$, ** $p < 0.01$

[a] Significantly higher CRP levels in females (after correction for multiple testing no longer significant)

[b] No longer significant after correction for multiple testing (Bonferroni)

Abbreviations: BMI=Body-Mass-Index; CRP= C-reactive protein; SII=systemic immune-inflammation index; NLR=neutrophil-to-lymphocyte ratio; MLR=monocyte-to-lmyphocyte ratio.

Appendix Table 4: Overview of changes between dates regarding the PANSS illness severity categories

Time interval	Category change	N	Percentage
Baseline - Week 2	Detoriation	2	0.94
Baseline - Week 2	Improvement	17	8.02
Baseline - Week 2	No Change	36	16.98
Baseline - Week 4	Improvement	4	1.89
Baseline - Week 4	No Change	10	4.72
Baseline - Week 8	Improvement	2	0.94
Baseline - Week 8	No Change	3	1.42
Baseline - Week 12	Improvement	1	0.47
Week 2 - Week 4	Detoriation	6	2.83
Week 2 - Week 4	Improvement	7	3.3
Week 2 - Week 4	No Change	29	13.68
Week 2 - Week 8	Improvement	1	0.47
Week 2 - Week 8	No Change	1	0.47
Week 2 - Week 12	Improvement	1	0.47
Week 2 - Week 12	No Change	1	0.47
Week 4 - Week 8	Detoriation	2	0.94
Week 4 - Week 8	Improvement	3	1.42
Week 4 - Week 8	No Change	18	8.49
Week 4 - Week 12	Detoriation	3	1.42
Week 4 - Week 12	Improvement	6	2.83
Week 4 - Week 12	No Change	8	3.77
Week 4 - Week 24	Improvement	1	0.47
Week 8 - Week 12	Detoriation	1	0.47
Week 8 - Week 12	Improvement	3	1.42
Week 8 - Week 12	No Change	21	9.91
Week 8 - Week 24	No Change	1	0.47
Week 12 - Week 24	Detoriation	4	1.89
Week 12 - Week 24	Improvement	4	1.89
Week 12 - Week 24	No Change	16	7.55
	Σ Improvement	50	23.59
	Σ Detoriation	18	8.49
	Σ No Change	144	67.93
	Σ Total	212	100.00

Improvement is associated with a change from a category indicating a more severe illness status to a category indicating a less severe illness status between measurements.
Detoriation is associated with a change from a category indicating a less severe illness status to a category indicating a more severe illness status between measurements.
No change is associated with no change between categories between measurements.

Categories are based on the differentiation provided by Leucht et al., 2005; DOI: https://doi.org/10.1016/j.schres.2005.04.008 with PANSS scores indicating the severity of illness: <= 58 “mildly ill”; 58 <= 75 “moderately ill”; 75 <= 95 “markedly ill”; 95 >= 116 "severely ill"; >116 "extremely ill"

Appendix Table 5: CRP descriptives in dependence of PANSS illness severity categories*

	PANSS Category*	Mean	SD	Median	Min	Max	N
Baseline	mildly ill	0.480	0.436	0.310	0.060	1.590	39
	moderately ill	0.282	0.291	0.100	0.060	1.130	29
	markedly ill	0.594	0.889	0.385	0.090	5.020	34
	severely ill	0.593	0.364	0.490	0.100	1.170	11
	extremely ill	0.335	0.219	0.335	0.180	0.490	2
	Total	0.472	0.583	0.280	0.060	5.020	115
Week 2	mildly ill	0.656	1.077	0.195	0.060	5.400	30
	moderately ill	0.525	0.607	0.180	0.060	1.970	23
	markedly ill	0.404	0.436	0.295	0.090	1.370	8
	severely ill	0.338	0.208	0.490	0.100	0.490	5
	Total	0.556	0.823	0.195	0.060	5.400	66
Week 4	mildly ill	0.495	0.455	0.280	0.060	1.980	39
	moderately ill	0.415	0.311	0.400	0.060	1.320	29
	markedly ill	0.507	0.478	0.360	0.100	1.580	17
	severely ill	0.694	0.769	0.490	0.100	2.040	5
	Total	0.482	0.437	0.375	0.060	2.040	90
Week 8	mildly ill	0.589	0.574	0.475	0.070	2.820	42
	moderately ill	0.331	0.271	0.240	0.100	0.950	12
	markedly ill	1.180	1.692	0.730	0.100	5.230	8
	severely ill	1.270	1.096	1.265	0.490	2.040	2
	Total	0.636	0.796	0.490	0.070	5.230	64
Week 12	mildly ill	0.583	0.701	0.385	0.100	3.530	30
	moderately ill	0.472	0.514	0.120	0.100	1.640	11
	markedly ill	0.653	0.183	0.615	0.490	0.890	4
	severely ill	0.200	0.141	0.200	0.100	0.300	2
	Total	0.546	0.615	0.390	0.100	3.530	47
Week 24	mildly ill	0.516	0.657	0.295	0.070	2.900	24
	moderately ill	0.532	0.488	0.490	0.100	1.890	11
	markedly ill	0.167	0.091	0.130	0.100	0.270	3
	Total	0.493	0.585	0.300	0.070	2.900	38

* Leucht et al., 2005; DOI: https://doi.org/10.1016/j.schres.2005.04.008

Appendix Table 6: Number of cases excluded at each time point due to CRP values indicating acute inflammation (CRP > 1 mg/dL).

	Total cases	Excluded cases (CRP > 1mg/dL)	% excluded*
Baseline	116	18	15.5
Week 2	57	15	26.3
Week 4	57	9	15.8
Week 8	26	1	3.9
Week 12	45	10	22.2
Week 24	26	6	23.1

* Percentages refer to the proportion of cases excluded relative to the total number of available cases at each assessment.

Appendix Table 7: Linear mixed-effects analysis investigating the association of changes in peripheral inflammatory marker levels and changes in PANSS scoring using different adjusted models

	Model		Estimate	S.E.	df	t	Estimate (95% CI)		*p-value*	AIC	BIC
							LB	UB			
CRP	Model 1	CRP	-6.059	3.841	167	-1.577	-13.642	1.524	0.1166	2002	2020
	Model 2	CRP (adjusted for sex & age)	-6.274	3.828	166	-1.639	-13.832	1.285	0.1032	2000	2025
	Model 3	CRP (adjusted for sex, age, & SS)	-6.348	3.852	162	-1.648	-13.955	1.258	0.1013	1941	1969
	Model 4	CRP (adjusted for sex, age, SS, & BMI)	-5.773	3.767	148	-1.532	-13.217	1.672	0.1276	1799	1830
SII	Model 1	SII	< -0.0001	0.001	151	0.172	-0.002	0.003	0.8636	1849	1866
	Model 2	SII (adjusted for sex & age)	< -0.0001	0.001	150	0.149	-0.002	0.003	0.8818	1846	1873
	Model 3	SII (adjusted for sex, age, & SS)	< -0.0001	0.001	147	0.181	-0.002	0.003	0.8564	1795	1822
	Model 4	SII (adjusted for sex, age, SS, & BMI)	< -0.0001	0.001	133	0.167	-0.002	0.003	0.8674	1654	1684
NLR	Model 1	NLR	0.008	0.352	151	0.022	-0.687	0.703	0.9822	1838	1855
	Model 2	NLR (adjusted for sex & age)	0.001	0.352	150	0.002	-0.695	0.697	0.9979	1837	1861
	Model 3	NLR (adjusted for sex, age, & SS)	0.002	0.353	147	0.005	-0.696	0.700	0.9957	1784	1811
	Model 4	NLR (adjusted for sex, age, SS, & BMI)	0.030	0.370	133	0.081	-0.701	0.761	0.9354	1643	1673
MLR	Model 1	MLR	0.625	2.119	151	0.295	-3.562	4.811	0.7686	1834	1851
	Model 2	MLR (adjusted for sex & age)	0.735	2.124	150	0.346	-3.462	4.931	0.7298	1834	1858
	Model 3	MLR (adjusted for sex, age, & SS)	0.707	2.125	147	0.334	-3.493	4.907	0.7399	1780	1807
	Model 4	MLR (adjusted for sex, age, SS, & BMI)	0.929	2.222	133	0.418	-3.467	5.325	0.6767	1639	1669

Abbreviations: AIC = Akaike Information Criterion; BIC = Bayesian Information Criterion; BMI = Body-Mass-Index; CRP = C-reactive protein; df = degree of freedom; LB=lower bound; MLR = monocyte-to-lymphocyte ratio; NLR = neutrophil-to-lymphocyte ratio; S.E. = standard error; SII = systemic immune-inflammation index; SS = Smoking status; UB=upper bound
